# Phases and Architectures in Metal/Metal Oxide Systems Driven by Strong Metal–Support Interactions

**DOI:** 10.1021/acsphyschemau.5c00138

**Published:** 2026-01-29

**Authors:** Jordi Morales-Vidal, Zan Lian, Thaylan Pinheiro Araújo, Sharon Mitchell, Javier Pérez-Ramírez, Núria López

**Affiliations:** † 202569 Institute of Chemical Research of Catalonia (ICIQ-CERCA), The Barcelona Institute of Science and Technology, Av. Països Catalans 16, Tarragona 43007, Spain; ‡ Institute of Chemical and Bioengineering, Department of Chemistry and Applied Biosciences, 27219 ETH Zurich, Vladimir-Prelog-Weg 1, Zurich 8093, Switzerland; § NCCR Catalysis, Zurich 8093, Switzerland

**Keywords:** Strong metal−support interactions, Interfaces, Reducible metal oxides, Density functional theory, Machine learning interatomic potentials

## Abstract

Metal oxide supported metal catalysts are widely applied in industrial processes. Many of these materials dynamically evolve under reducing atmospheres, leading to metal nanoparticles partially or fully encapsulated by metal oxide shells, impacting catalytic performance. This phenomenon is known as strong metal–support interaction (SMSI) and is thermodynamically driven. However, understanding the metal/metal oxide interfaces derived from the broad and flexible compositional space and the large structural changes in SMSI structures is difficult to monitor experimentally. Here, we use density functional theory together with machine learning interatomic potentials and global minima optimization to investigate SMSI by building a set of interfaces between common catalytic metals (Ni, Pd, Pt) and reducible metal oxides (r-TiO_2_, CeO_2_, In_2_O_3_) at different reduction levels. Phase diversity arises from the competition between the formation of different metal oxides or binary alloys, while the local properties of the suboxide layers are responsible for the final architecture and composition determining the electronic properties of the material. Two descriptors related to the competition between alloy and oxide formation are proposed to elucidate the phase diversity. Our work provides a systematic approach to advance the design of SMSI-based catalytic materials by offering insights into the atomic-level architecture of the metal/metal oxide interfaces.

## Graphical abstract

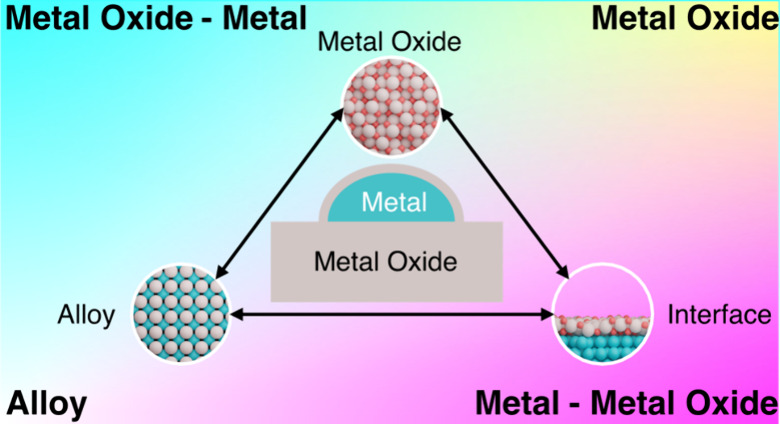

## 1 Introduction

Supported metal catalysts have been widely used in catalytic chemistry, particularly in hydrogenation reactions.^1–5^ However, the traditional view that metal oxide supports behave as inactive carriers to disperse and enhance the utilization of metal species, considered as the active phase, has been challenged since the 70s.^6,7^ Indeed, metal–support interactions (MSI) play a crucial role in determining the stability of supported metal catalysts and enhancing their activity.^8–16^ MSI has been assigned to electronic coupling between the metal and the metal oxide support (creation of the Schottky barriers),^17–19^ enabling bifunctional mechanisms via the contribution from the metal/metal oxide periphery,^20–22^ responsible for changes in the morphology of nanoparticles,^23–26^ and changes in the composition of the metal nanoparticles (NPs).^27–29^

A particular case of MSI are strong metal–support interactions (SMSI) that appear when a system prepared as metal NPs deposited on a metal oxide are exposed to a reductive reaction environment and evolves to generate thin suboxide layers (1 to 3 partially reduced layers), partially or completely encapsulating the metal nanoparticle.^5–7,30–36^ SMSI was first reported by Tauster et al. for Group VIII noble metals supported on TiO_2_.^6^ It was observed that the adsorption of CO and H_2_ molecules was inhibited after high temperature reduction but could be restored upon exposure to oxidative atmospheres. Since then, various characterization techniques (Auger electron spectra, X-ray photoelectron spectroscopy, X-ray absorption fine structure spectroscopy, high-resolution electron microscopy, and gas chemisorption) have been employed to assess encapsulation.^37^

SMSI has been extensively documented for catalysts comprising metal NPs supported on TiO_2_,^5,38–45^ CeO_2_,^33,46–56^ and In_2_O_3_ ^21,29,32,57^ carriers. As these catalytic systems are often employed in CO and CO_2_ hydrogenation reactions, their performance is directly impacted by SMSI, which alters the chemisorption properties of small molecules such as CO, CO_2_, and H_2_. Moreover, SMSI is frequently invoked to account for enhanced activity in metal oxide interfaces and deemed to increase nanoparticle stability.^58^

CO_2_ hydrogenation is a clear example of the role of SMSI in catalytic performance. Although Ni possesses intrinsic methanation activity,^29,59^ SMSI induces changes in selectivity. In Ni/CeO_2_, ceria enhances the conversion to methane due to the dynamic formation of Ce^3+^ active sites at the interface with Ni NPs.^46,59^ In contrast, a partially reduced patchy TiO_2_ overlayer provides interfacial Ni sites favoring carbon–carbon coupling as C_2+_ hydrocarbons.^5,55^ In turn, Ni/In_2_O_3_ systems boost methanol formation rather than CO or CH_4_, due to In–Ni alloyed layers that facilitate H_2_ splitting and oxygen vacancy formation on In_2_O_3_.^29^ Instead, in a closely related system (the ternary Pd-In_2_O_3_/*m*-ZrO_2_ catalyst), the strong interaction of In_2_O_3_ with Pd under reaction conditions leads to the migration of InO*_*x*_* suboxides patches that partially cover Pd NPs and form InPd*_*x*_* alloys.^21^ This SMSI-derived architecture yields a stable catalyst that integrates acid–base and redox functions, promoting the hydrogenation of CO_2_ to methanol.

SMSI also leads to electron transfer from TiO_2_ to Pt, boosting selective catalytic reduction of NO*_*x*_* by promoting the reagents adsorption and reactivity.^44^ Moreover, partial encapsulation of Pt NPs by CeO_2_ support has been found critical for promoting propane dehydrogenation rather than hydrogenolysis.^48^ This is due to the partial covering of extensive Pt ensembles that are active for propane hydrogenolysis. For Pd NPs, SMSI with CeO_2_ enhances catalytic oxidation of ethyl acetate to CO_2_ by generating surface oxygen vacancies and activated oxygen species.^47^ Finally, on different metal-supported NPs, discontinuous nondense support overlayers of CeO_2_ or TiO_2_ SMSI-derived were found using ultrafast laser to boost CO oxidation increasing the availability of active sites at the metal–support interface.^33^

Thus, despite the huge fundamental and practical impact of SMSI-derived structures in catalysis, interrogating the complex metal/metal oxide interfaces is experimentally difficult. Advanced microscopy techniques have confirmed the encapsulation of metal NPs by the support, but intrinsic limitations of these methods restrict their scope.^31,60^ Furthermore, *in situ* measurements have shown that the structure dynamically changes with the environments instead of behaving in a static manner once formed.^5,21,38,40,46^

The driving force for SMSI is to minimize the surface energy of the total system that is high for the metal and low for the reducible metal oxide.^33,49,61–63^ Most density functional theory (DFT) studies that address the interfacial structures employ pristine metal oxide or hydroxy oxide layers.^64–68^ Instead, machine learning techniques^69^ fitted to experimentally determined interfacial adhesion energies between NPs and metal oxide supports have proposed that SMSI arises from the competition between the metal–metal bonding energy of the supported metal and the metal oxide compared to the metal of the metal oxide.^70^ Still, an atomic-level understanding of the metal/metal oxide interfaces and the role of suboxide formation and its degree of reducibility remains elusive.

Here, we have used DFT and machine learning interatomic potentials (MLIPs) coupled to global optimization to study SMSI formation in systems integrating a common catalytic metal (Pd, Pt, or Ni) on a reducible metal oxide carrier (CeO_2_, rutile-TiO_2_, and In_2_O_3_). Both the individual components and the interface are modeled to map and identify descriptors for the preferred architectures under a reducing atmosphere. The overall SMSI architecture is dominated by the competition between the metal–metal bond strength and metal–oxygen bond strength; the degree of reduction of the suboxide depends on the support, and the composition at the interface depends on the metal and the metal oxide.

## 2 Results and Discussion

### 2.1 Modeling SMSI

Nine different metal/metal oxide systems were selected to assess SMSI via DFT and MLIP coupled to global minima optimization (Figure 1). All DFT simulations were performed with the Vienna *ab initio* simulation package (VASP) by means of Perdew–Becke–Ernzerhof (PBE) functional, including the Hubbard *(U*) parameter for highly correlated *d* or *f* electrons of metal oxides (r-TiO_2_, NiO, and CeO_2_).^71–74^ Further information on the computational details can be found in Methods and Supporting Methods (Figure S1 and Tables S1–S5).

**Figure 1:**
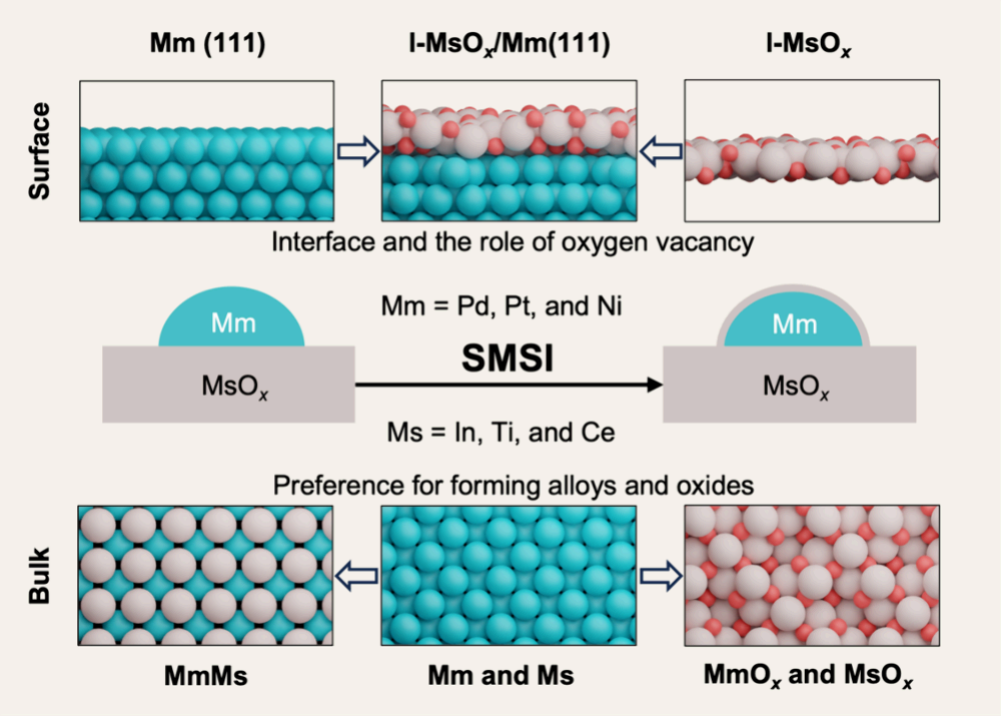
Modeling approach to understand SMSI in Mm/MsO_*x*_ systems. Common metal/metal oxide interfaces were modeled by generating systems combining representative metal (Mm = Pd, Pt, or Ni) and metal oxide layers at different reduction degrees (l-MsO_*x*_, Ms = In, Ti, or Ce). Bulk models were used to compare the affinity of the six metals to form single-phase metals, metal oxides, or alloys, covering a total of 57 systems. The surface models were employed to assess the interface and the role of oxygen vacancies. 228 configurations were used to explore the stability of the different interfaces at different degrees of reduction of the metal oxide layers, which can be viewed as a proxy of considering different reductive environments.

We started with a pool of three face-centered cubic (fcc) metals (Mm = Pd, Pt, and Ni) and the metals corresponding to the support (Ms = Ce, Ti, and In). This phase space was then expanded with the most stable metallic and metal oxide phases, as well as with MmMs alloys following the structures in Materials Project database.^75^ For all these materials, the bulk energies were obtained, and for Mm and the most stable metal oxides of Ms the surface energies were evaluated (Table S1). For the (111) termination of the fcc metals we employed four-layered slabs representing the (111) termination. While for the metal oxides (MsO_*x*_), the most stable polymorph and the lowest surface energy orientations were considered, rutile for TiO_2_(110), cubic fluorite for CeO_2_ (111), and cubic bixbyite for In_2_O_3_ (111). The corresponding slabs contain different number of trilayers (O-Ms-O).

### 2.2 SMSI and Metal/Metal Oxide Interfaces

The reduction of the overall surface energy of metal/metal oxide systems via metal encapsulation is the commonly accepted driving force for SMSI.^33,49,61–63^ Therefore, we started by computing the surface energy (γ_surf_) of the individual metal and metal oxide phases (Table S1). Our results align with experimental observations, as we retrieved smaller values for the pristine reducible metal oxides (γ_surf, MsO*x*_ < 0.049 eV/Å^2^) than for the metals (γ_surf, Mm_ > 0.083 eV/Å^2^). Finally, the energy required to form a monolayer of the metal oxide from the slabs is double their associated surface energies, since they form 2 surfaces (Table S1). To promote encapsulation, this energy would need to be compensated by forming metal/metal oxide bonds.

Then, we assessed the stability (*E*, eqs 1 and 2) of metal oxide layers (N-MsO_*x*_, N = 1–3) covering the (111) termination of Pd, Pt, and Ni. The interface was built by keeping the lattice parameters of the metals and adapting the supercell of the oxide to that of the metal. This leads to mismatch between 2 and 10% on a and b lattice parameters of the metal oxide (Table S2), while the resulting strain is partially alleviated by the formation of adatoms or patches, especially following global optimization. We only considered a single layer of MsO*_*x*_* since the interaction of the first layer with metallic surfaces is in general higher than that of multiple layers of the metal oxide (Table S3). Moreover, the deposition of one layer is favored over two and three (Table S4). Figure 2 indicates that the process of depositing a metal oxide layer (with respect to the bulk metal oxide) on the metal is endothermic for all systems in the fully oxidized form (0 degree of reduction of l-MsO*_*x*_*). Therefore, there is no wetting of the pristine metal oxide layers on Pd, Pt, and Ni, so metal and metal-oxides are separated under normal (oxidic) conditions.

**Figure 2:**
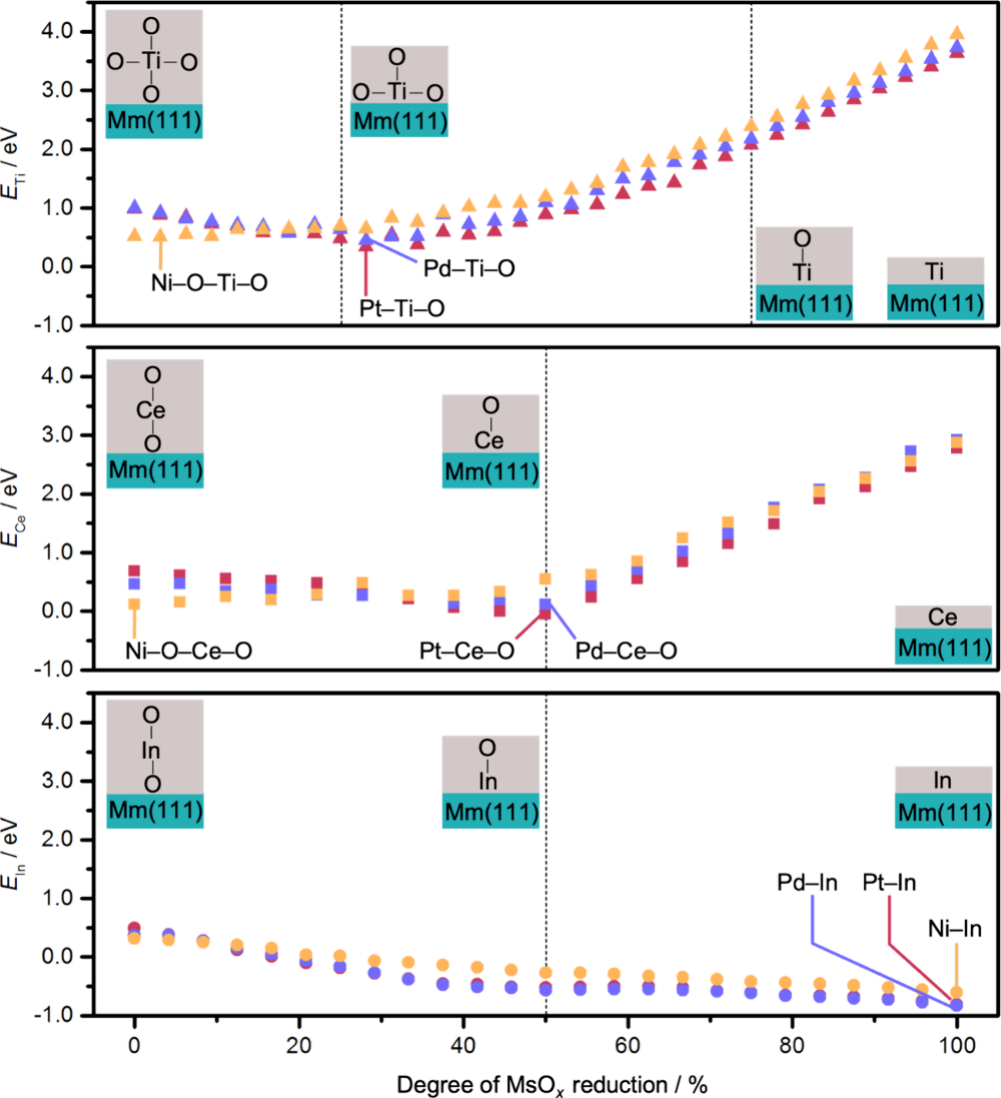
Stability of the MsO_*x*_/Mm systems at different reduction degrees by means of the DFT-optimized structures. The stability is measured as the potential energy (*E*, eV) with H_2_, H_2_O, and bulk metal oxides as references (eqs 1 and 2). The schemes illustrate relevant models at different degrees of reduction separated by dashed lines and the most stable for each case indicated. The most stable structure for each MsO_*x*_/Mm system is shown in Figure S7. The insets are schematic representations employed to simplify the complex structure. Both DFT and MLIP simulations reveal that oxygen atoms at the interface are less stable and they are preferentially removed first.

When reducible metal oxides are exposed to environments containing H_2_ or CO, they tend to form oxygen vacancies more easily than the other metal oxides. We investigated first how the monolayers of r-TiO_2_, CeO_2_, and In_2_O_3_ respond to increasing degrees of reduction. Figure S2 shows that the three reducible oxides behave in a rather different manner. The vacancy formation energy of In_2_O_3_ steadily decreases as the system is more reduced. For r-TiO_2_ and CeO_2_ vacancy formation is first favored, leading to the most stable structures at 17% and 22% reduction degree, respectively. Vacancies in these materials are linked to the formation of polarons and it is well-known that this creates repulsion as the emerging M^3+^ cations repel each other.^76^ This driving force hinders the deep reduction of the oxide layers, making the process endothermic at higher degrees of reduction.

Besides, since the interfaces can exhibit a flexible compositional space and large structural changes, we employed a fine-tuned MLIP model coupled to minima hopping to assess the robustness of the structures obtained via DFT.^77^ The database (73047 structures) including interfaces optimized with DFT were used to fine-tuned foundation model of MACE-MPA-0 medium architecture MLIP model (see Methods).^78^ For the MLIPs, the root-mean-square error (RMSE) of the energies is 6.8 meV/atom on the training set and 7.7 meV/atom on the validation set, while the RMSE of the forces are 28.0 and 27.5 meV/Å on the training and validation sets, respectively. The differences in energies are much lower than the chemical accuracy, 43 meV/Å, which indicates that the MLIP model has reached the accuracy with respect to the reference DFT level. Then, the fine-tuned MLIP model was employed to run 100 molecular dynamics and 101 optimizations with minima hopping algorithm,^79^ using the DFT-optimized structures of the interfaces at different degree of reductions as the initial configuration. To avoid errors in the model in the extrapolation area, we computed a single point with DFT of the minima hopping-MLIP proposed minima structures. The comparison of the single point energies and MLIP energies for the new minima gives a RMSE range from 3.09 to 33.34 meV/atom for different systems (Figure S4). The DFT energies of the minima identified via minima hopping are generally lower than those of the DFT-optimized interface structures (Figure S5). However, the stability trends of the interfaces across different degrees of reduction are consistent between the minima hopping and DFT-optimized structures (Figure 2 and Figures S6–S8). This indicates that the DFT-optimized structures are robust and representative of the systems under study.

For In_2_O_3_, reduction improves the adhesion of the suboxide to the metal. Adsorption of the reduced CeO_2_ and r-TiO_2_ layers is slightly less favorable. These suboxide monolayers stick more strongly than pristine ones to Pd and Pt, in contrast to Ni that prefers oxide monolayers with larger oxygen contents. The most stable structures of Pd and Pt appear at low oxygen vacancy concentration for r-TiO_2_ (29%), and at slightly higher reduction degree (50%) for CeO_2_. This trend follows the patterns identified for the oxygen vacancy formation in isolated oxide layers, but in all cases shifting the optimal values to higher reduction degrees (Figure 2 and Figure S2). The higher reduction degree observed for CeO_2_ is consistent with previous works indicating that higher reduction temperatures are required for CeO_2_ compared to oxide, and the energy required to detach the suboxide monolayer is compensated by its interaction with the metallic surfaces, which makes the metal wetting by the suboxide possible (Figure S3). In turn, the most stable architecture at the interface depends on both the metal and the metal oxide (Figure 2 and Figures S6–S8), which are key for catalytic performance. Fully oxidized r-TiO_2_ and CeO_2_ with Ni lead to Mm-O-Ms-O patterns, while the suboxide Mm-Ms-O structures are observed for Pd and Pt. In_2_O_3_ exhibits reduced structures with In-Mm interactions for all three metals.

Charge transfer processes induced by SMSI influence both the activity and selectivity patterns in catalysis.^17,32,44,46,47,53,55^ Therefore, we evaluated how the electronic properties of Ni, Pd, Pt, Ti, Ce, and In are modified upon adsorption of pristine and the most stable suboxide layers on Mm. We calculated the averaged shift of the Bader charges of metals (Δ*q*_Mm_, Figure 3, Table S6) in the outermost layer of the metal slab after interacting with metal oxide layers. Figure 3 shows that Mm atoms can be oxidized or reduced depending on the degree of reduction of the metal oxide layer. For the pristine layers (0% MsO*_*x*_* reduction), Mm atoms are oxidized while they are reduced for suboxides (*X*% MsO*_*x*_* reduction). Thus, the direction of the charge transfer between the metal and metal oxides changes as a function of reduction of metal oxide layers, while its intensity depends on the nature of the Mm atoms. The Bader charges of the rest of the Mm in the slabs are almost unaffected (Table S7), while the average charge of the cations (Ms) in the oxide layers (Figure S9 and Table S8) follows the degree of reduction (Figure S2).

**Figure 3:**
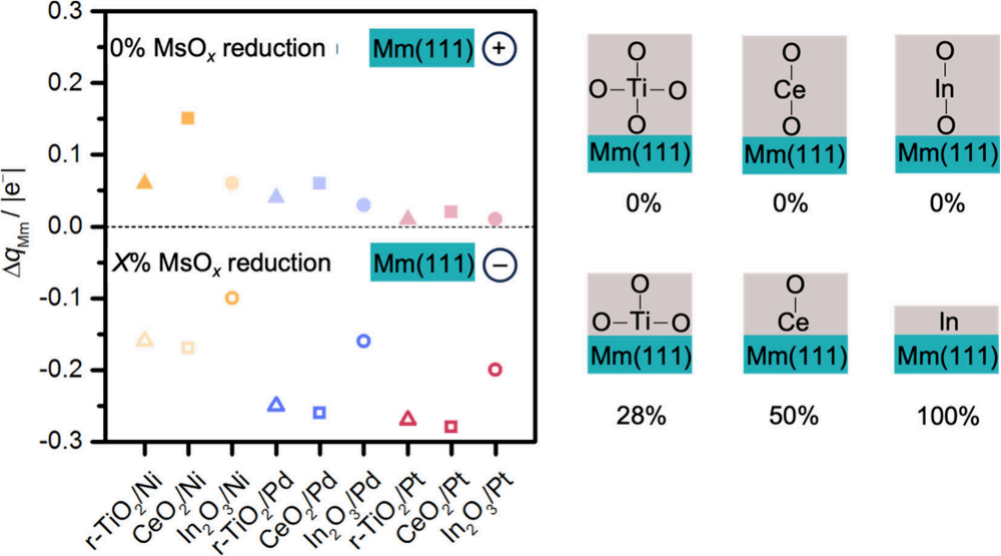
Electronic properties of Ni, Pd, and Pt atoms at the interface with MsO_*x*_ layers. Δ*q*_Mm_ is the shift in the average Bader charges of the outermost atoms of the Mm(111) slabs interacting with the reduced MsO_*x*_ layers with respect to that of the isolated Mm(111) slabs. Filled and unfilled symbols indicate 0 and *X*% MsO_*x*_ reduction degree, respectively, where *X*% is indicated in the schemes. Semitransparent data points represent structures that are not the most stable systems.

### 2.3 Descriptors to Elucidate the Architecture of the Interface

Some metal oxides (i.e., In_2_O_3_) can become fully reduced, triggering the formation of metal–metal bonds and thus alloy phases in the metal particles. To assess the relative stability of competing phases, we evaluated different bulk alloys and binary metal oxides containing all metals considered Pd, Pt, Ni and Ce, Ti, In. Particularly, we proposed the Δ*E*_Mm_ and Δ*E*_Ms_ descriptors (competition between forming alloys or oxides for each Ms and Mm, eqs 3 and 4) to assess the tendency of each of the six metals to form an alloy versus a metal oxide (Figure 4, Tables S5, S9, and S10, details in Methods and Supporting Methods). When doing so, the phase diagram contains four extreme regions encompassing nine possible architectures (Figure 4 and Figure S10). This spans from fully metallic behavior, with alloys dominating at the bottom left, to oxophilic character and oxide–oxide compositions at the top right.

**Figure 4:**
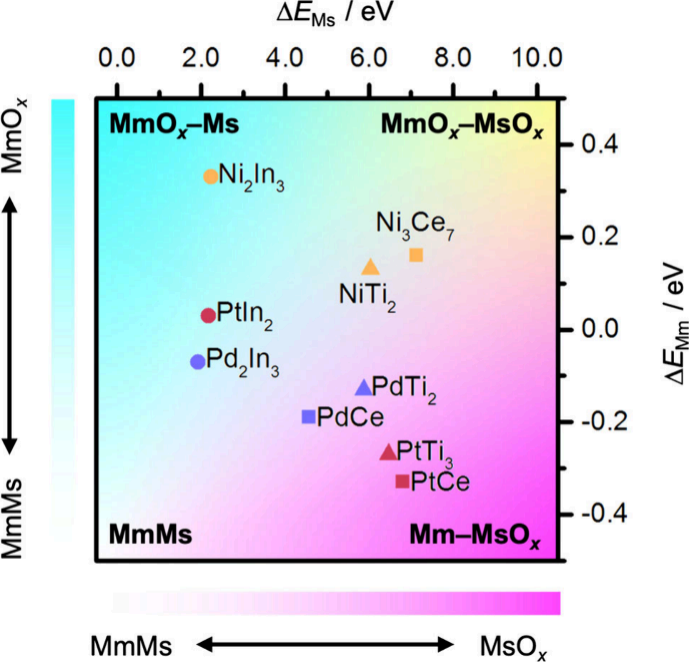
Classification of system architectures. Δ*E*_Ms_ and Δ*E*_Mm_ are descriptors for the competition of Ms or Mm to form a metal oxide or an alloy, respectively. Structural extremes are marked in bold, and the complete classification of regions is shown in Figure S10.

Based on Δ*E*_Mm_ and Δ*E*_Ms_ descriptors, a different behavior is found for In compared to Ce and Ti, and between the noble metals (Pd and Pt) and Ni (Figure 4). The relative positions of the systems in the diagram are also in line with the results obtained for the interface between the metal oxides and metal accounting for their most stable systems (Figure 2). Specifically, systems with Pd and Pt are at the bottom of the diagram (Mm-Ms), whereas systems with Ni are found at the top side (Mm-O-Ms) due to the higher affinity of the latter toward oxygen. The systems containing In fall at the left side of the diagram as they form strong Mm–Ms bonds, while Ce or Ti systems are found at the right side due to their higher oxidic character (Mm-Ms-O).

The elucidation of these trends enables a rational understanding of the types of interfacial interactions that emerge in SMSI-dominated systems. It should be noted that this work is based on global minima structures and zero temperature energetics. Therefore, additional entropic and dynamic contributions must be considered to predict specific SMSI-derived structures under the given reaction conditions. Here, we provide a thermodynamic and classificatory framework to rationalize the SMSI architectures of different catalytically relevant materials. The interfacial interactions define both the chemical nature of the interfacial motifs and the spatial distribution of the active sites, which are critical for catalyst design. The proper combination of different functionalities in spatially resolved sites facilitates efficient reagent activation while minimizing long-range sluggish and stochastic transport. The higher activity in CO_2_ hydrogenation for the Ni/CeO_2_ system^46^ can be qualitatively understood as the closer proximity between the metal and the support that being more basic is able to better activate CO_2_. In turn, on the TiO_2_ support,^5,55^ probably related to the less amount of oxygen vacancies (5% compared to 20–50% for ceria) in the oxide film that allows the stabilization of radicals for C_2+_ product formation. Finally, the identification of alloy-type structures in the In_2_O_3_ systems is line with previous observations for Ni and Pd on In_2_O_3_ where such structures were found key for boosting CO_2_ hydrogenation to methanol.^21,29,57,80^

Therefore, a holistic understanding of SMSI requires the integration between the competition of the bulk phases (metal oxides versus alloys) and the understanding of reduced suboxide monolayers at the interface. The degree of reduction of the metal oxide controls the interaction at the interface of the metal oxide with the metal, which governs the wetting properties. Furthermore, the nature of the metals in supports and metal NPs dictates the stoichiometry and the pattern of the final SMSI-induced architectures. The diverse interfacial motifs identified across different types of metals (Ni and the noble Pt and Pd) with three metal oxides with varying reducibility (TiO_2_, CeO_2_, and In_2_O_3_), provide insights for the rational design of catalysts. The specific architecture and chemical nature of the interfacial patterns (whether metallic, oxidic, acid–base, and/or redox active, as well as the spatial distribution of the sites) critically determine the catalytic activity, selectivity, and stability of these SMSI-dominated systems.

## 3 Conclusion

We have used DFT simulations and MLIP coupled to minima hopping to investigate, at the atomistic level, the interface architecture of metals supported on metal oxides under reductive conditions relevant to key catalytic transformations including hydrogenations and related reactions involving complex redox dynamics. Our findings indicate that general energy considerations govern strong metal–support interactions. In particular, the resulting phases and interface structures can be understood by evaluating the stability of the individual bulk phases relative to their binary combinations. For each Ms (metal in oxide phase) and Mm (metal in metallic phase), the competition between alloy and metal oxide formation, quantified by Δ*E*_Mm_ and Δ*E*_Ms_, successfully served as descriptors distinguishing the different SMSI behaviors. When the primary catalytic metal becomes encapsulated, the reducibility patterns of the metal oxide determine the oxygen content, while the proximity of oxygen to the interface also depends on the metal type. These aspects collectively modulate the charge transfer at the interfaces. Thus, we have elucidated the governing principles of interface formation and the derived architectures in a generalizable framework. This paves the way toward rational design of active, selective, and robust metal/metal oxide catalysts with tailored interfaces.

## 4 Methods

Density functional theory (DFT) simulations were carried out with Vienna *ab initio* simulation package (VASP 5.4.4).^71,72^ The functional of choice was Perdew–Becke–Ernzerhof (PBE)^73^ functional corrected with the Hubbard (*U*) parameter^74^ according to Dudarev’s approach^81^ to mitigate the self-interaction error of metal oxides with high correlated *d* or *f* electrons (r-TiO_2_, NiO, and CeO_2_).^82^ In line with previous works, we used *U* = 4.2 eV and *U* = 5.3 eV for *d* electrons of Ti and Ni, respectively, while we employed *U* = 4.5 eV for *f* electrons of Ce.^83–85^ We used plane-waves with a kinetic cutoff energy of 500 eV to describe the valence electrons whereas core electrons were represented with projector augmented-wave (PAW) core potentials.^86,87^ Nevertheless, we employed a kinetic cutoff energy of 700 eV to optimize the bulk lattice parameters of different metals, alloys, and metal oxides comprising Ms (In, Ce, and Ti) and Mm, (Pd, Pt, and Ni). The Brillouin zone was sampled with a Γ-centered mesh with a reciprocal grid size narrower than 0.033 2π·Å^–1^ generated by means of the Monkhorst–Pack method.^88^ Spin polarization was included when needed.

Machine learning interatomic potential was fine-tuned to enable the assessment of the DFT-optimized interface structures by the minima hopping algorithm. The model was fine-tuned using MACE code based on the foundation model of MACE-MPA-0 medium,^78^ which gave the architecture of 128 × 0e + 128 × 1o. The data set contains 73047 structures, including 13 bulks, 114 structures from slab relaxation, 61230 systems from interface relaxation, and 11690 structures from metal oxide layer relaxation. The data set was randomly divided into training and validation sets in the ratio of 95:5. A learning rate of 0.0001 was used. Minima hopping algorithm^79^ built in the Atomic Simulation Environment (ASE) package^89^ was used to search the minima for the interface structures. 600 K was used for the initial temperature and 100 steps were run.

The potential energy (*E*) associated with a metal oxide layer at different degrees of reduction (l-MsO*_*x*_*, where Ms = In, Ti, or Ce) deposited on slab models of the (111) termination of different face-centered cubic metals (Mm = Pd, Pt, or Ni) was obtained with eqs 1 and 2 (further details can be found in Supporting Methods). *E* was employed to assess the driving force leading to the encapsulation of the metals by metal oxide layers or the formation of alloys under reductive conditions. Considering different degrees of reduction can be viewed as a proxy for different reductive environments. The bulk of the metal oxides (*E*_MsO_*x*_, bulk_^DFT^), molecular hydrogen (*E*_H_2_, gas_^DFT^), and water (*E*_H_2_O, gas_^DFT^) were employed as references and the parameter *v* stands for the number of oxygen vacancies. Although structural relaxations are allowed in these simulations, no lattice optimization is allowed. All of the energies are computed per Ms atom.

$$\mathrm{Ms}O_{x}+\mathrm{Mm}+vH_{2}\to \mathrm{l‐Ms}O_{x-v}/\mathrm{Mm}+vH_{2}O$$ 1

$$E=E_{l-\mathrm{Ms}O_{x-v}/\mathrm{Mm}}^{\mathrm{DFT}}+vE_{H_{2}O,\mathrm{gas}}^{\mathrm{DFT}}-E_{{\mathrm{MsO}}_{x},\mathrm{bulk}}^{\mathrm{DFT}}-E_{\mathrm{Mm},\mathrm{slab}}^{\mathrm{DFT}}-vE_{H_{2},\mathrm{gas}}^{\mathrm{DFT}}$$ 2

We explored the tendency of each of the six metals (Ms = In, Ti, Ce and Mm = Pd, Pt, Ni) to form an alloy (MmMs) or a metal oxide (MO*_*x*_*) to assess the competition between alloy and metal oxide formation. To this end, we used Δ*E*_Mm_ and Δ*E*_Ms_ descriptors obtained with eqs 3 and 4 (further details can be found in Supporting Methods). *y* represents the number of Ms atoms in the MmMs alloy and the term 1+*y* is the total number of atoms in MmMs employed to obtain a normalized Δ*E*_Mm_ and Δ*E*_Ms_ with respect to a unit of alloy for each system. *E*_MmMs_y__ stands for the energy required for one atom of Mm and *y* atoms of Ms in their metallic bulk to form the MmMs alloy (eqs S1 and S2). *E*_MmO*x*_ and *E*_MsO*x*_ are the energies required for one atom of Mm or Ms to form its associated metal oxide (eqs S3–S6).

$$\Delta E_{\mathrm{Mm}}=\left(E_{\mathrm{Mm}{\mathrm{Ms}}_{y}}-E_{\mathrm{Mm}O_{x}}\right)/\left(1+y\right)$$ 3

$$\Delta E_{\mathrm{Ms}}=\left(E_{\mathrm{Mm}{\mathrm{Ms}}_{y}}-E_{\mathrm{Ms}O_{x}}\right)/\left(1+y\right)$$ 4

## Supplementary Material



## Data Availability

DFT and MLIPs data can be found online in the ioChem-BD repository^90,91^ at http://dx.doi.org/10.19061/iochem-bd-1-325.

## Notes

Supporting methods with extended details of the methodologies and approaches employed in this work, supporting tables including computational details of each simulated system and associated values, and supporting figures including sketches, snapshots, and properties of the simulated systems, results of the fine-tuned MLIP used in this work, and extended phase diagram of the SMSI architectures (PDF)
