# Supplementary material for: Phases and Architectures in Metal/Metal Oxide Systems Driven by Strong Metal–Support Interactions
Source: ACS Phys Chem Au. 2026 Jan 29;6(2):340–7. doi: 10.1021/acsphyschemau.5c00138 (PMC13022790; doi:10.1021/acsphyschemau.5c00138)
Supplement: Supplementary file 1 [file pg5c00138_si_001.pdf]

Supporting Information

Phases and Architectures in Metal/Metal Oxide Systems Driven by Strong Metal-Support Interactions

Jordi Morales-Vidal,[1] Zan Lian,[1] Thaylan Pinheiro Araújo,[2] Sharon Mitchell,[2,3] Javier Pérez-Ramírez,[2,3] and Núria López[1]**

[1]Institute of Chemical Research of Catalonia (ICIQ-CERCA), The Barcelona Institute of Science and Technology, Av. Països Catalans 16, Tarragona 43007, Spain

[2]Institute of Chemical and Bioengineering, Department of Chemistry and Applied Biosciences, ETH Zurich, Vladimir-Prelog-Weg 1, Zurich 8093, Switzerland

[3]NCCR Catalysis, Zurich 8093, Switzerland

Table of Contents

1. Supporting Methods 3

2. Supporting Tables 6

3. Supporting Figures 16

4. Supporting References 26

1. Supporting Methods

1.1 Adsorption of l-MsO_x on Mm(111) Surfaces

The potential energy (*E*) associated with a metal oxide layer at different degrees of reduction (l-MsO_x, where Ms = In, Ti, or Ce) deposited on slab models of the (111) termination of different face-centered cubic metals (Mm = Pd, Pt, or Ni) was obtained with **Equations 1** and **2**. *E* was employed to assess the driving force leading to the encapsulation of the metals by metal oxides layers or the formation of alloys under reductive conditions. To this end, we optimized a monolayer of each metal oxide on top of a slab model of each metal. Then, we evaluated the *E* associated with the optimized structures obtained by removing oxygen by oxygen of the metal oxide layer (from the closest oxygen atoms to the farthest from the metal surface) until reaching a completely reduced layer (**Figure S1a**). We also tried to remove first a layer of oxygen atoms situated farthest from the interface (*i.e.,* interacting with the vacuum). However, after relaxation, the remaining oxygen atoms in the interface in contact with the metal moved upwards to occupy the positions of the removed oxygen atoms in all systems except In_2O_3/Ni (**Figure S1b**). Thus, the oxygen atoms in the interface are less stable than the ones in the surface, and the interfacial oxygen atoms would be first reduced from the thermodynamic standpoint. Furthermore, we employed a fine-tuned machine learning interatomic potential model coupled to minima hopping to assess the optimized structures obtained *via* density functional theory (DFT).

Regarding the slab models of the metals, we employed different reconstructions of the (111) surface of Pd, Pt, and Ni to maintain the commensurability with the metal oxide layers that completely cover the metal surface (**Table S2**). The slab models contained four atomic layers where the two bottommost were fixed to their bulk positions and the two outermost were allowed to relax.

For the metal oxide layers, we employed a O-M-O tri-layer obtained from a slab model of the most stable termination of In_2O_3, CeO_2 and rutile-TiO_2 (**Table S1**).[1–3] The In_2O_3 layer was built from a *p*(1×1) slab of the (111) termination of the cubic bixbyite structure. The CeO_2 layer was obtained from a *p*(3×3) reconstruction of the (111) surface of the cubic fluorite structure. Finally, we built a r-TiO_2 layer from a *p*(4×2) slab of the (110) termination of the rutile polymorph. We used a O-M-O tri-layer of the three metal oxides to assess the interaction between these metal oxides layers at different degrees of reduction and the (111) termination of the face-centered cubic metals. The formation of oxygen vacancies in CeO_2 and r-TiO_2 is associated with polaron formation and leads to repulsive interactions, as the resulting M^{3+}

cations repeal each other. The stability of these systems as a function of vacancy concentration can be influenced by the model size due to periodic interactions. Thus, the dimension of the layers used for CeO_2 and r-TiO_2 are comparable to ensure similar periodic polaron-polaron interactions. Furthermore, the interaction of l-MsO_x at 0% degree of reduction with the metals was found in general higher or of the same magnitude than with a second oxide layer for the 9 systems (**Table S3**). Therefore, the formation of monolayers of r-TiO_2, CeO_2, and In_2O_3 adsorbed on the metals is favored over multilayer structures when oxide supply is limited. Moreover, we evaluated the deposition of two and three layers of the metal oxide on Pd, Pt, and Ni. The adsorption of one layer is favored over two and three **(Table S4**).

1.2 Competition Between Alloy and Metal Oxide Formation

We assessed the tendency of each of the six metals (Ms = In, Ti, and Ce; Mm = Pd, Pt, Ni) to form an alloy (MmMs) or a metal oxide (MO_x). Firstly, we optimized the lattice parameters of the different metal bulks, alloys, and metal oxides. For the metal bulks we employed the most stable structures of each metal. Therefore, face-centered cubic structures (space group number 225) were used for Pd, Pt, and Ni. Ti and Ce were represented with hexagonal close-packed structures (space group number 194). Finally, the tetragonal structure from the space group number 139 was employed for In.

In reference to the MsMm alloys, we optimized the lattice parameters for the 5 most stable bulks from Materials Project,[4] which have been experimentally reported. Then we computed the energy required for one atom of Mm in its metallic bulk to form each alloy following (E_{MmMs}, **Equations S1** and **S2)** and we selected the most favored structures (**Table S9**). We used the criteria of one atom of Mm in its metallic bulk instead of one atom of Ms since the amount of Mm is clearly smaller than Ms in the systems selected to explore the strong metal-support interactions (metals supported on metal oxides). In such a way, y is the number of Ms atoms in MmMs.

$$\mathrm{Mm} + y\mathrm{Ms} \rightarrow \mathrm{MmMs}_y \tag{S1}$$

$$E_{\mathrm{MmMs_y}} = E^{\mathrm{DFT}}_{\mathrm{MmMs_y,\,bulk}} - E^{\mathrm{DFT}}_{\mathrm{Mm,\,bulk}} - yE^{\mathrm{DFT}}_{\mathrm{Ms,\,bulk}} \tag{S2}$$

Regarding the metal oxides, we optimized the bulk of the thermodynamically most stable structures.[5,6] We used the cubic bixbyite structure of In_2O_3 (space group number 206), the cubic fluorite for CeO_2 (space group number 225), the rutile polymorph of TiO_2 with a tetragonal symmetry (space group number 141), the tetragonal PdO with space group number 131, the PtO_2 from the orthorhombic crystal system (space group number 158), and the cubic NiO (space

group number 225). With these structures at hand, we computed the energy required for one atom of Mm or Ms to form its associated oxide using a molecule of oxygen as reference (E_{MmO_x} and E_{MsO_x}, **Table S5, Equations S3-S6**).

$$\mathrm{Mm} + \frac{x}{2}\mathrm{O_2} \rightarrow \mathrm{MmO}_x \qquad \text{S3}$$

$$E_{\mathrm{MmO_x}} = E^{\mathrm{DFT}}_{\mathrm{MmO_x,\ bulk}} - E^{\mathrm{DFT}}_{\mathrm{Mm,\ bulk}} - \frac{x}{2} E^{\mathrm{DFT}}_{\mathrm{O_2,\ gas}} \qquad \text{S4}$$

$$\mathrm{Mm} + \frac{x}{2}\mathrm{O_2} \rightarrow \mathrm{MsO}_x \qquad \text{S5}$$

$$E_{\mathrm{MsO_x}} = E^{\mathrm{DFT}}_{\mathrm{MsO_x,\ bulk}} - E^{\mathrm{DFT}}_{\mathrm{Ms,\ bulk}} - \frac{x}{2} E^{\mathrm{DFT}}_{\mathrm{O_2,\ gas}} \qquad \text{S6}$$

Then, the competition between forming alloys or oxides for each Ms and Mm (ΔE_{Ms} and ΔE_{Ms}, respectively) was obtained with **Equations 3** and **4** (**Table S10**).

2. Supporting Tables

Table S1. Termination, supercell, number of layers employed to build slab models of metals and metal oxides to compute their associated surface energies (γ_{surf} / eV·Å^{-2}), and energy required to detach one layer of metal oxide from its surface ($E_{l\text{-}MsOx}$ / eV·Å^{-2}).

System	Termination	Reconstruction	Layers (fixed+relaxed)	γ_{ssurf}[a] / eV·Å^{-2}	$E_{l\text{-}MsOx}$[b] / eV·Å^{-2}
Pd	(111)	$p(5\times5)$	2+2	0.083	-
Pt	(111)	$p(5\times5)$	2+2	0.091	-
Ni	(111)	$p(6\times6)$	2+2	0.120	-
r-TiO_2	(110)	$p(4\times2)$	3+2[c]	0.049	0.211
CeO_2	(111)	$p(3\times3)$	1+2[d]	0.045	0.101
In_2O_3	(111)	$p(1\times1)$	3+2[e]	0.048	0.097

[a] g_{surf} was calculated from equation $\gamma_{surf} = \frac{1}{2A}(E_{slab,\,unrelax} - N_{slab}E_{bulk}) + \frac{1}{A}(E_{slab,\,relax} - E_{slab,\,unrelax})$ where A is the area of the surface; $E_{slab,\,unrelax}$ and $E_{slab,\,unrelax}$ represent the energy of the surface as cleaved from its optimized bulk and the energy of the optimized surface, respectively; N_{slab} stands for the number of atoms in the slab; and E_{bulk} is the energy associated with one atom in the bulk.

[b] $E_{l\text{-}MsOx}$ was calculated from equation $E_{l\text{-}MsOx} = (E_{l\text{-}MsOx} + E_{MsOx,\,slab-1} - E_{MsOx,\,slab})/A$ where $E_{l\text{-}MsOx}$ is the energy of a layer of the metal oxide, $E_{MsOx,\,slab}$ stands for the energy of a slab of the metal oxide, $E_{MsOx,\,slab-1}$ is the energy associated with a slab of the metal oxide with a detached layer, and A represents the surface area of the slab.

[c] Each r-TiO_2 layer is formed by 3 atomic layers (O-Ti-O).

[d] Each CeO_2 layer is formed by 3 atomic layers (O-Ce-O). The oxygen atoms of the middle layer that are in contact with the bottommost layer were also fixed to their bulk positions.

[e] Each In_2O_3 layer is formed by 3 atomic layers (O-In-O).

Table S2. Supercells of the (111) termination of the Pd, Pt, and Ni and reconstructions of r-TiO_2 (110), CeO_2(111), and In_2O_3(111) layer employed to compute the stability of the interface (*E*, **Equations 1 and 2**), and the % of strain induced on the a (a_{strain}) and b (b_{strain}) lattice parameters of the metal oxide layers by adapting its supercell to the one of the metals.

System	Metal supercell	Metal oxide supercell	a_{strain}[a]	b_{strain}[b]
r-TiO_2/Pd	*p*(4×6)	*p*(4×2)	–7.81	9.65
r-TiO_2/Pt	*p*(4×6)	*p*(4×2)	–7.18	10.40
r-TiO_2/Ni	*p*(5×6)	*p*(4×2)	2.91	–2.08
CeO_2/Pd	*p*(4×4)	*p*(3×3)	–4.45	–4.45
CeO_2/Pt	*p*(4×4)	*p*(3×3)	–3.80	–3.80
CeO_2/Ni	*p*(5×5)	*p*(3×3)	6.66	6.66
In_2O_3/Pd	*p*(5×5)	*p*(1×1)	–4.32	–4.32
In_2O_3/Pt	*p*(5×5)	*p*(1×1)	–3.67	–3.67
In_2O_3/Ni	*p*(6×6)	*p*(1×1)	2.53	2.53

[a] % of strain was calculated from equation $a_{strain} = \frac{a_{Mm} - a_{MsO_x}}{a_{Mm}} \cdot 100$, where a_{MsO_x} is the lattice parameter a of the surfaces of the oxides (**Table S1**) and a_{Mm} is the lattice parameter b of the (111) termination of the face-centerd cubic metals.

[b] % of strain was calculated from equation $b_{strain} = \frac{b_{Mm} - b_{MsO_x}}{b_{Mm}} \cdot 100$, where b_{MsO_x} is the lattice parameter b of the surfaces of the oxides (**Table S1)** and b_{Mm} is the lattice parameter b of the (111) termination of the face-centered cubic metals.

Table S3. Interaction energy normalized by the number of metal atoms (Ms = Ti, Ce, or In) in the metal oxides layers ($E_{\text{int, Ms}}$ / eV) between two metal oxide layers (l-MsO_x/l-MsO_x) or one metal oxide layer and a metallic surface (l-MsO_x/Pd, l-MsO_x/Pt, and l-MsO_x/Ni). $E_{\text{int, Ms}}$ for l-MsO_x/l-MsO_x was computed with $E_{\text{int, Ms}} = E^{\text{DFT}}_{\text{2l-MsO}_x} - 2E^{\text{DFT}}_{\text{l-MsO}_x}$, where $E^{\text{DFT}}_{\text{l-MsO}_x}$ and $E^{\text{DFT}}_{\text{2l-MsO}_x}$ are the energies associated with one and two layers of the metal oxide layers, respectively. $E_{\text{int, Ms}}$ for the systems with one metal oxide layer at 0 degree of reduction deposited on the three face-centered cubic metals was obtained with **Equations 1** and **2** using a layer of the metal oxides instead of the respective bulks as references.

	$E_{\text{int, Ms}}$ / eV			
MsO_x	l-MsO_x/l-MsO_x	l-MsO_x/Pd	l-MsO_x/Pt	l-MsO_x/Ni
TiO_2	–0.56	–0.73	–0.61	–0.78
CeO_2	–0.73	–0.86	–0.64	–1.19
In_2O_3	–1.71	–1.05	–1.07	–1.35

Table S4. Adsorption energy ($E_{ads, Ms}$ / eV) normalized by the number of metal atoms (Ms = Ti, Ce, or In) associated with the deposition of one, two, and three metal oxide layers on Pd, Pt, and Ni (111) surfaces.

	$E_{ads, Ms}$ / eV		
System	1-layer	2-layer	3-layer
r-TiO_2/Pd	–1.05	0.43	0.40
r-TiO_2/Pt	–1.07	0.42	0.29
r-TiO_2/Ni	–1.53	0.28	0.72
CeO_2/Pd	–0.86	–0.26	–0.13
CeO_2/Pt	–0.64	–0.20	–0.10
CeO_2/Ni	–1.19	0.10	0.13
In_2O_3/Pd	–0.73	–0.27	–0.12
In_2O_3/Pt	–0.61	–0.24	–0.11
In_2O_3/Ni	–0.78	–0.30	–0.20

Table S5. Formation energy of different metal oxides (E_{MOx} / eV) normalized by the number of metal atoms and obtained with **Equations S3-S6** and experimental standard enthalpy of formation ($\Delta_f H^o_{MOx}$ / eV).

Metal oxide	E_{MOx} / eV	$\Delta_f H^o_{MOx}$ [5,6] / eV
TiO_2	–9.48	–9.78
CeO_2	–10.49	–11.28
In_2O_3	–3.99	–4.80
PdO	–0.97	–0.89
PtO_2	–1.52	–1.71
NiO	–1.23	–2.48

Table S6. Averaged shift in the Bader charges for the atoms on the outermost atomic layer of the Mm(111) slabs, due to the interaction with the MsO_x layers at different degrees of reduction, with respect to the averaged Bader charges of the isolated metal slabs (Δq_{Mm} / |e⁻|). The degrees of reduction (% red.) associated with *X* are shown in the chemical schemes of **Figure 3**.

% red.	l-MsO_x	Mm		
		Ni	Pd	Pt
0	r-TiO_2	0.06	0.04	0.01
	CeO_2	0.15	0.06	0.02
	In_2O_3	0.06	0.03	0.01
X	r-TiO_2	–0.16	–0.25	–0.27
	CeO_2	–0.17	–0.26	–0.28
	In_2O_3	–0.10	–0.16	–0.20

Table S7. Averaged shift in the Bader charges for the atoms on subsurface of the Mm(111) slabs, due to the interaction with the MsO_x layers at different degrees of reduction, with respect to the averaged Bader charges of the isolated metal slabs (Δq_{Mm} / $|e^-|$). The degrees of reduction (% red.) associated with X are shown in the chemical schemes of **Figure 3**. These values indicate that the magnitude of the charge transfer of subsurface metallic layers and MsO_x layers are significantly smaller than for the atoms in the outermost layer of Mm(111) slabs. In the case of the bottommost layers, we found $\Delta q_{Mm} < 0.01$ $|e^-|$.

% red.	l-MsO_x	Mm		
		Ni	Pd	Pt
0	r-TiO_2	–0.01	–0.03	–0.04
	CeO_2	–0.02	–0.03	–0.03
	In_2O_3	–0.01	–0.03	–0.03
X	r-TiO_2	–0.02	–0.05	–0.06
	CeO_2	–0.01	–0.04	–0.04
	In_2O_3	–0.01	–0.04	-0.05

Table S8. Averaged shift in the Bader charges of Ms on the MsO_x layers at different degrees of reduction and interacting with the Mm(111) slabs, with respect to the averaged Bader charges of the pristine isolated metal oxide layers (Δq_{Ms} / $|e^-|$). The degrees of reduction (% red.) associated with *X* are shown in the chemical schemes of **Figure 3**. As expected, the presence of Ti, Ce, or In atoms in the reduced MsO_x layers present always a negative $\Delta q_{Ms.}$

% red.	l-MsO_x	Mm		
		Ni	Pd	Pt
0	r-TiO_2	–0.01	0.00	0.02
	CeO_2	–0.32	–0.10	–0.06
	In_2O_3	–0.18	–0.07	–0.05
X	r-TiO_2	–0.18	–0.10	–0.07
	CeO_2	–0.53	–0.55	–0.49
	In_2O_3	–1.47	–1.47	-1.40

Table S9. Formation energy of the different metal alloys (E_{MmMs} / eV) normalized by the number of Mm atoms and obtained with **Equations S1** and **S2**. Mm represents Pd, Pt or Ni, while Ms stands for Ti, Ce or In. The most stable alloys are indicated in bold, which were used to measure the competition of each of the six metals to form an alloy or a metal oxide from their metallic bulks (ΔE_{Ms} and ΔE_{Ms}, **Table S10**).

Alloy	E_{MmIn} / eV	Alloy	E_{MmCe} / eV	Alloy	E_{MmTi} / eV
PdIn	–0.97	Pd_3Ce_7	–1.26	**$PdTi_2$**	**–1.35**
**Pd_2In_3**	**–1.15**	**PdCe**	**–1.35**	Pd_3Ti_2	–1.00
Pd_2In	–0.76	Pd_3Ce	–1.10	Pd_5Ti_3	–0.98
Pd_3In	–0.59	Pd_4Ce_3	–1.28	Pd_2Ti	–0.94
Pd_3In	–0.60	Pd_5Ce	–0.62	Pd_3Ti	–0.87
**$PtIn_2$**	**–1.42**	Pt_4Ce_3	–1.92	**$PtTi_3$**	**–2.59**
Pt_3In_2	–0.79	**PtCe**	**–2.18**	Pt_5Ti_3	–1.51
Pt_2In_3	–1.16	Pt_3Ce	–1.30	PtTi	–1.86
Pt_3In_7	–1.40	Pt_2Ce	–1.52	Pt_3Ti	–1.15
PtIn	–0.90	Pt_2Ce_3	–2.11	Pt_8Ti	–0.50
**Ni_2In_3**	**–0.41**	NiCe	–0.68	**$NiTi_2$**	**–0.85**
$Ni_{13}In_9$	–0.21	Ni_2Ce	–0.59	Ni_4Ti_3	–0.74
NiIn	–0.39	Ni_3Ce	–0.54	NiTi	–0.81
Ni_3In	–0.10	Ni_5Ce	–0.43	Ni_3Ti	–0.65
Ni_3In	–0.07	**Ni_3Ce_7**	**–0.69**	NiTi	–0.80

Table S10. Competition between forming alloys or oxides for Pd, Pt, and Ni (ΔE_{Mm} / eV) and for Mm (ΔE_{Ms} / eV) with respect to a unit of alloy for each system and obtained with **Equations 3** and **4**.

System	ΔE_{Ms} / eV	ΔE_{Mm} /eV
Pd_2In_3	2.41	–0.09
$PtIn_2$	2.18	0.03
Ni_2In_3	2.23	0.33
PdCe	4.57	–0.19
PtCe	6.81	–0.33
NiCe	6.02	0.22
$PdTi_2$	5.87	–0.13
$PtTi_3$	6.46	–0.27
$NiTi_2$	6.04	0.13

3. Supporting Figures

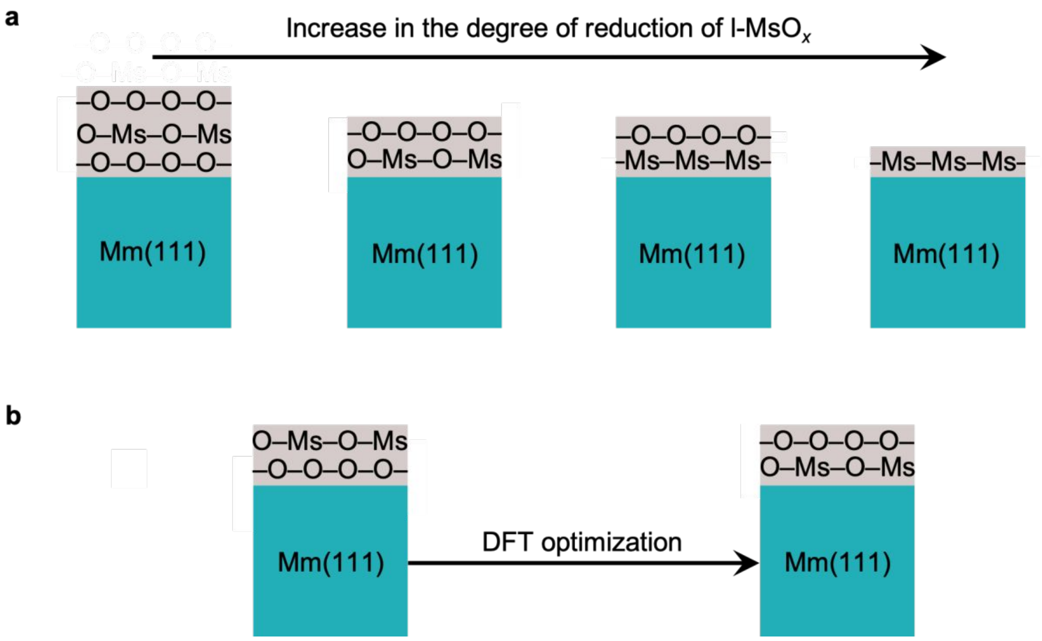

Figure S1. Sketch of the models of the interfacial structures at different degrees of reduction. (**a**) Selected structures of the metal oxide layer (l-MsO$_x$) deposited on the (111) termination of Mm at different degrees of reduction, indicating the sequence followed to remove oxygen atoms from the l-MsO$_x$. (**b**) General structural evolution evidenced by DFT optimization when trying to remove the layer of oxygen atoms from l-MsO$_x$ situated furthest from the interface (*i.e.,* interacting with vacuum). This behavior was not observed for In_2O_3/Ni due to the oxophilicity of nickel. Nevertheless, the structure with the oxygen atoms between indium and nickel is 0.20 eV less stable per In atom than the analogous structure without oxygen atoms in the interface. The oxygen atoms of the metal oxide layers that are closer to the metal surface are easier to remove since the metal-metal (Mm-Ms) interaction is favored over metal-oxygen (Mm-O) interaction in all systems but r-TiO_2 and CeO_2 Ni-containing systems (**Figure 2** and **Figure 4**). Furthermore, we have evaluated the Bader charges of the oxygen atoms of the metal oxide layers deposited on the metal surfaces**.** The results shows that the oxygen atoms in direct contact with the metal surface exhibit slightly more positive Bader charges than the other oxygen atoms in the metal oxide layer.

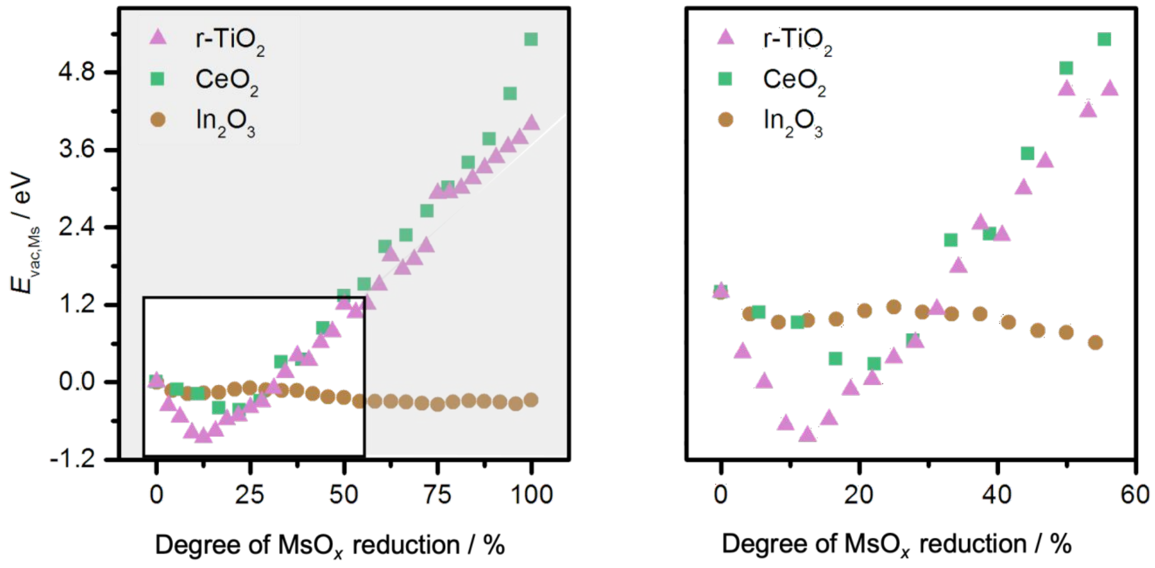

Figure S2. Vacancy formation energy for isolated layers of the metal oxides. The vacancy formation energies were simulated as l-MsO_x + vH_2 → l-MsO_{x-v} + vH_2O, and the energies were calculated by $E_{vac} = E^{DFT}_{l\text{-}MsO_{x-v}} + vE^{DFT}_{H_2O,\ gas} - E^{DFT}_{l\text{-}MsO_x} - v\mathrm{E}^{DFT}_{H_2,\ gas}$ taking into account one atom of Ms. Triangle, square, and circle correspond to TiO_x, CeO_x, and InO_x, respectively. Note that these oxides would pass through a few phase transformations when the degree of reduction increases but for simplicity these are not considered here.[7] Therefore, we only focus on the results associated with vacancy formation energy until 60% degree of reduction.

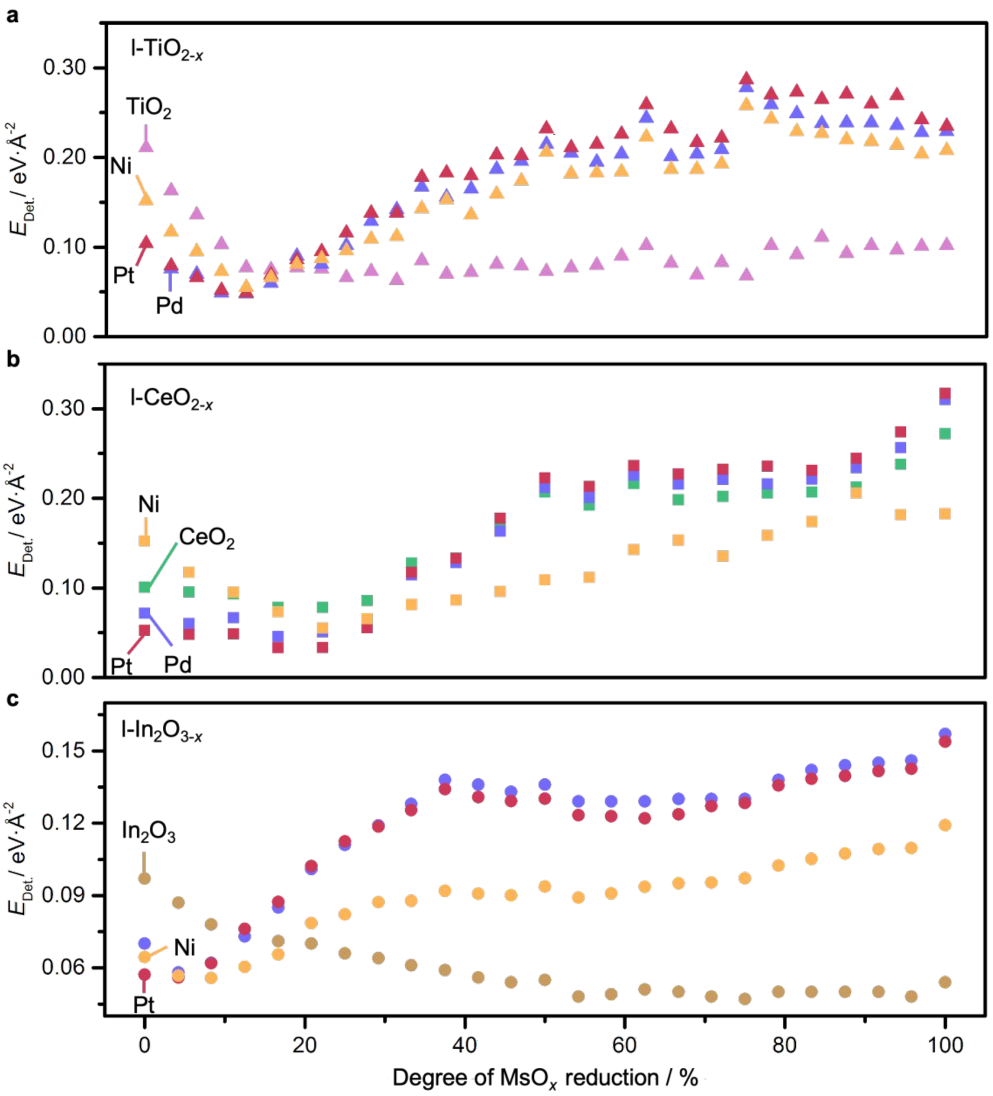

Figure S3. Wettability of l-MsO_x layers on different surfaces. Energy required to detach a layer ($E_{Det.}$ / eV·Å^{-2}) of r-TiO_2 (**a**), CeO_2 (**b**), and In_2O_3 (**c**) at different degrees of reduction from their associated metal oxide surfaces and from Pd, Pt, and Ni surfaces (**Table S1**). E_{Det} from the metal oxides (MsO_x) was calculated with the following equation $E_{Det} = (E_{l\text{-}MsOx} + E_{MsOx,\ slab-1} - E_{MsOx,\ slab})/A$, where $E_{l\text{-}MsOx}$ is the energy of a layer of the metal oxide at different degrees of reduction, $E_{MsOx,\ slab}$ stands for the energy of a slab of the metal oxide at different degrees of reduction, $E_{MsOx,\ slab\ -1}$ is the energy associated with a slab of the metal oxide with a detached layer, and A represents the surface area of the slab. In the case of E_{Det} from the metallic surfaces (Mm), we employed the following equation $E_{Det} = (E_{l\text{-}MsOx} + E_{Mm,\ slab} - E_{l\text{-}MsOx/Mm})/A$, where $E_{Mm,\ slab}$ stands for the energy of a slab of the metallic surfaces and $E_{l\text{-}MsOx/Mm}$ is the energy associated with a metal oxide layers at different degrees of reduction deposited on a metallic surface.

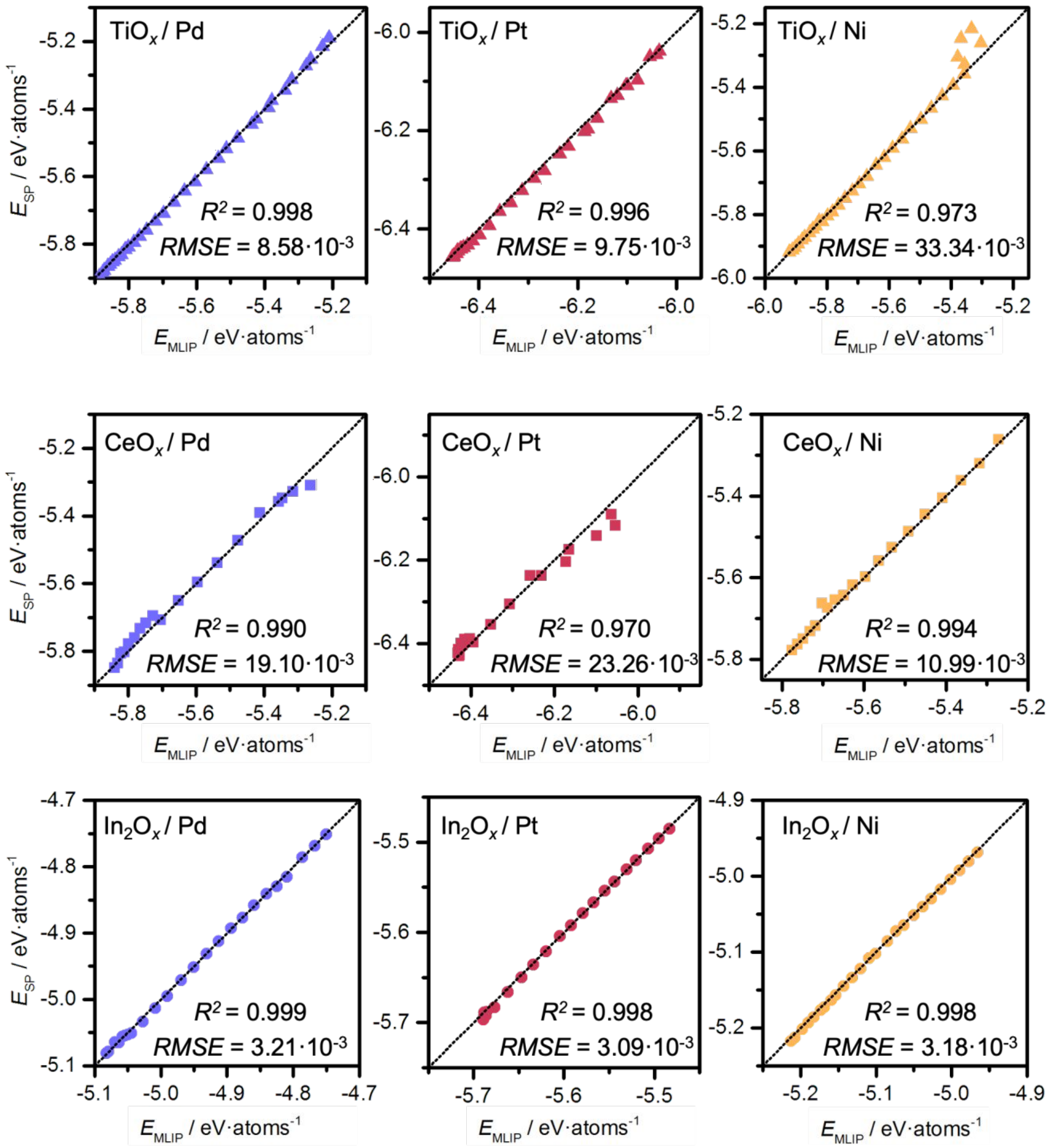

Figure S4. Parity plots of the energy associated with the interface structures at different degrees of reduction obtained by means of the fine-tuned MLIP starting from the DFT-optimized structures and running 100 steps of minima hopping (E_{MLIP}), and the energy obtained by performing a single point with DFT of these structures (E_{SP}). Triangles, squares, and circles correspond to CeO_x/Mm, TiO_x/Mm, and InO_x/Mm, respectively, while orange, blue, and red stands for MsO_x/Ni, MsO_x/Pd, and MsO_x/Pt, respectively.

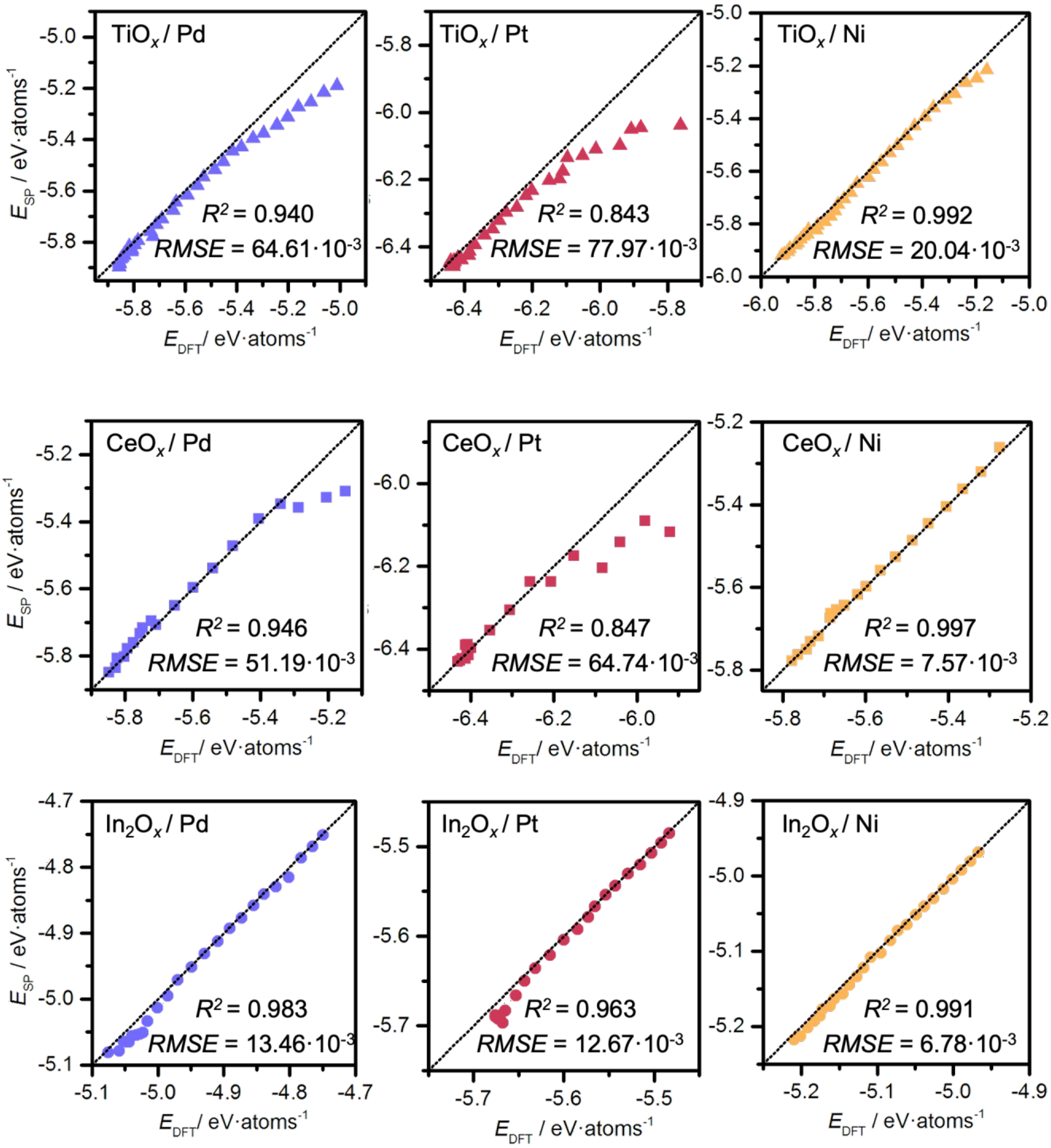

Figure S5. Parity plots of the energy associated with the interface structures at different degrees of reduction obtained by means of DFT-optimized structures (E_{DFT}), and the energy associated with the single point performed in the analogues structures obtained via the fine-tuned MLIP model coupled to minima hopping (E_{SP}). Triangles, squares, and circles correspond to CeO$_x$/Mm, TiO$_x$/Mm, and InO$_x$/Mm, respectively, while orange, blue, and red stands for MsO$_x$/Ni, MsO$_x$/Pd, and MsO$_x$/Pt, respectively. TiO$_x$/Pd, TiO$_x$/Pt, CeO$_x$/Pd, and CeO$_x$/Pt show points that deviates from the parity line for systems with more than 75% reduction degree. This is because the local optimized structures preserve a highly reduced metal oxide layer deposited on top of the metal surface, whereas the global optimalization leads to alloy formation between the highly reduced metal oxide layers and the metal surfaces.

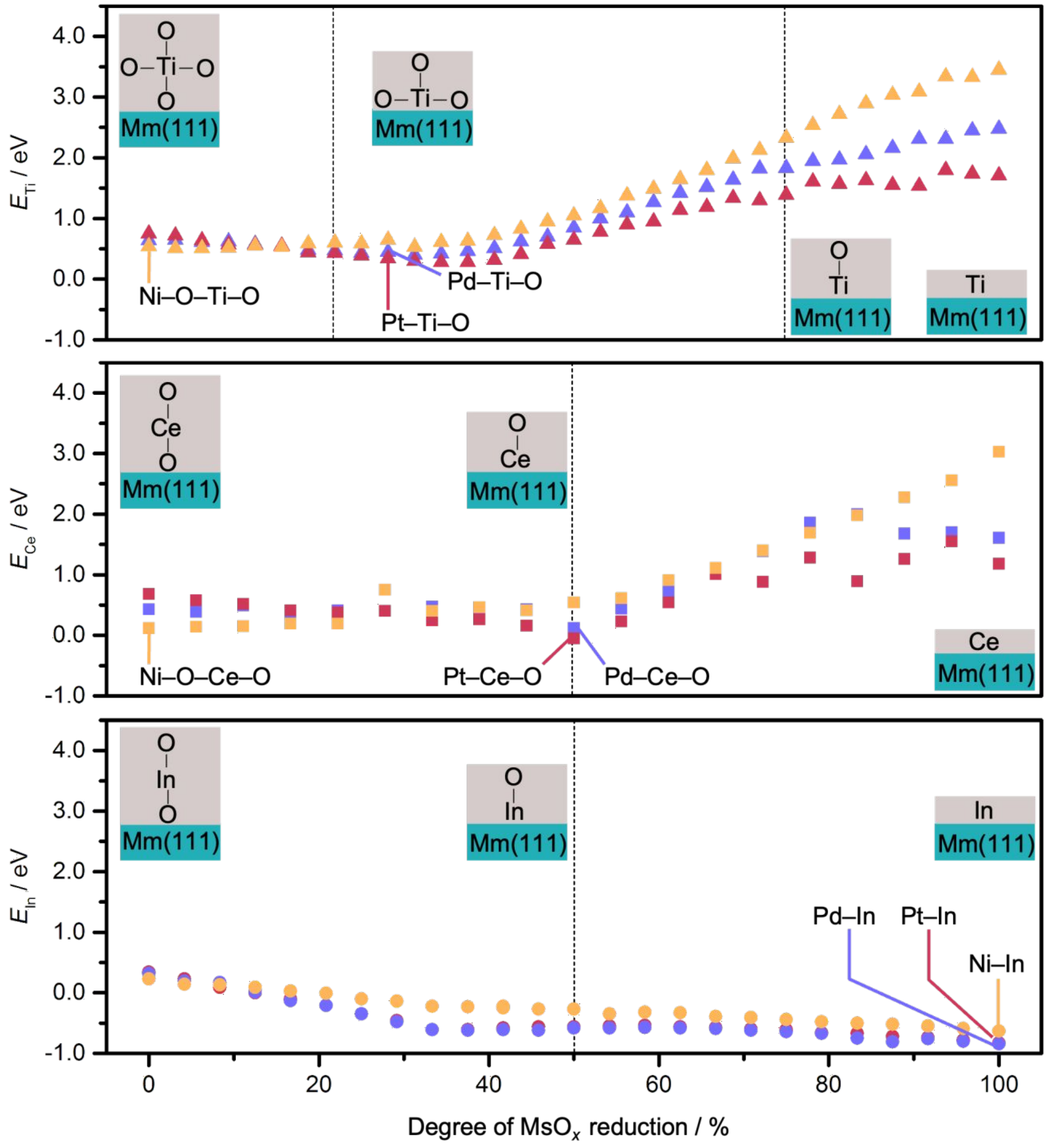

Figure S6. Stability of the MsO_x/Mm systems at different reduction degree by means of the structures obtained with the fine-tuned MLIP coupled to minima hopping and computing a single point with DFT to obtain the associated energy. The stability is measured as potential energy (E / eV) with H_2, H_2O, and bulk metal oxides as references (**Equations 1** and **2**). The schemes illustrate relevant models at different degrees of reduction separated by dashed lines and the most stable for each case indicated.

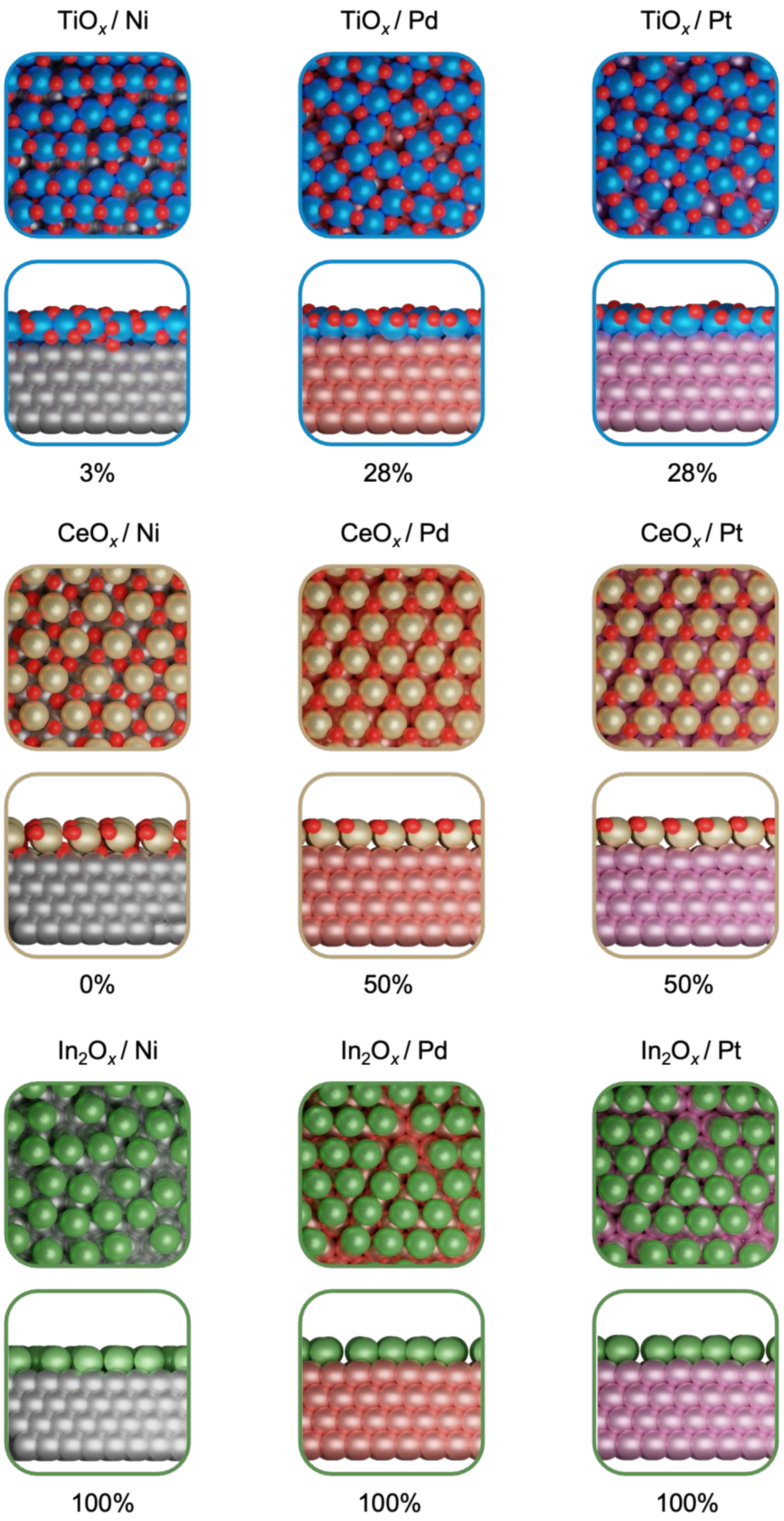

Figure S7. Top and side views of the most stable structures of the MsO_x/Mm systems at different reduction degree identified with DFT and associated with **Figure 2**. Color code: Ti (blue), Ce (pale yellow), In (green), Ni (gray), Pd (light pink), and Pt (Purple).

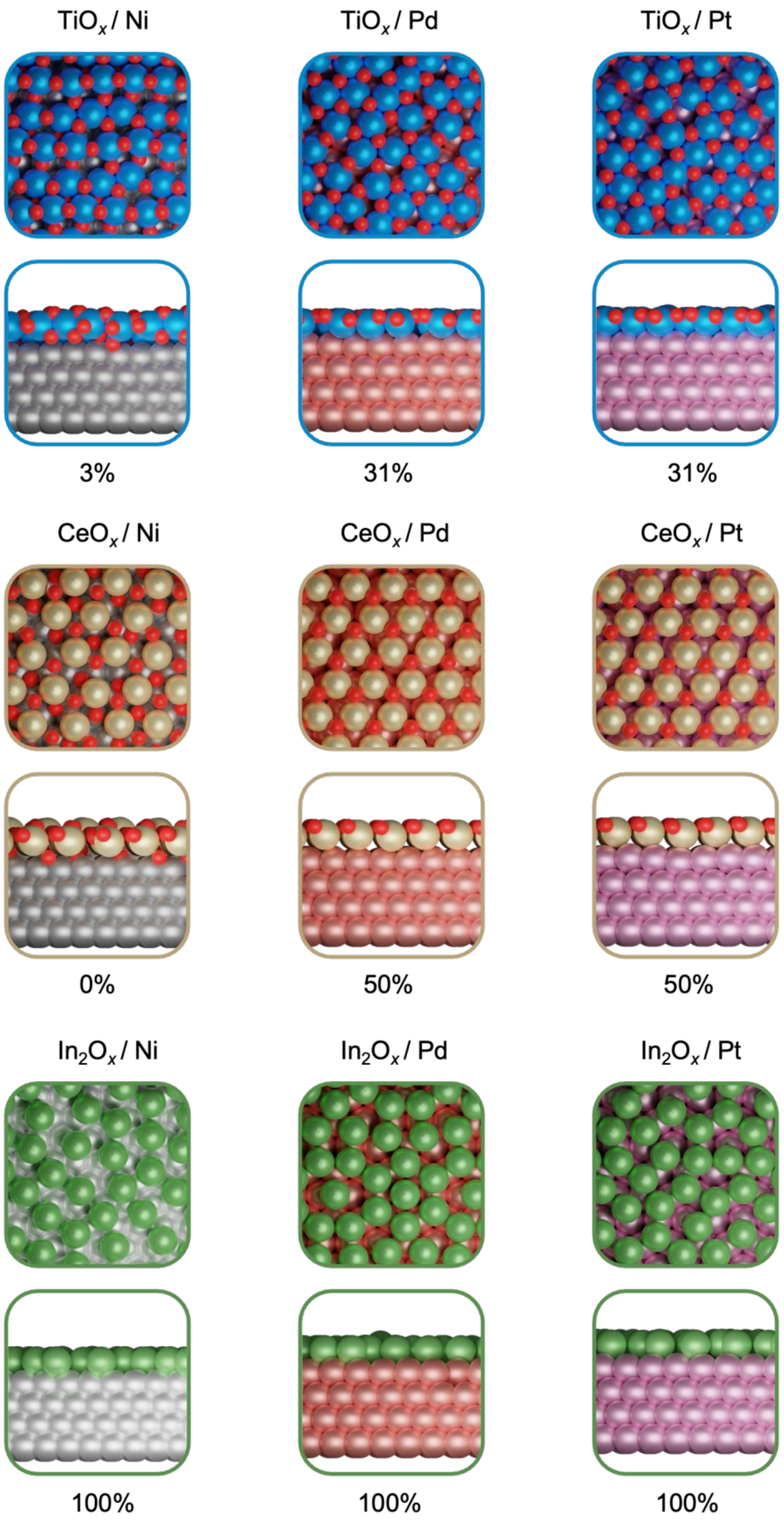

Figure S8. Top and side views of the most stable structures of the MsO_x/Mm systems at different reduction degree identified with the fine-tuned MLIP coupled to minima hopping and associated with **Figure S6**. Color code: Ti (blue), Ce (pale yellow), In (green), Ni (gray), Pd (light pink), and Pt (Purple).

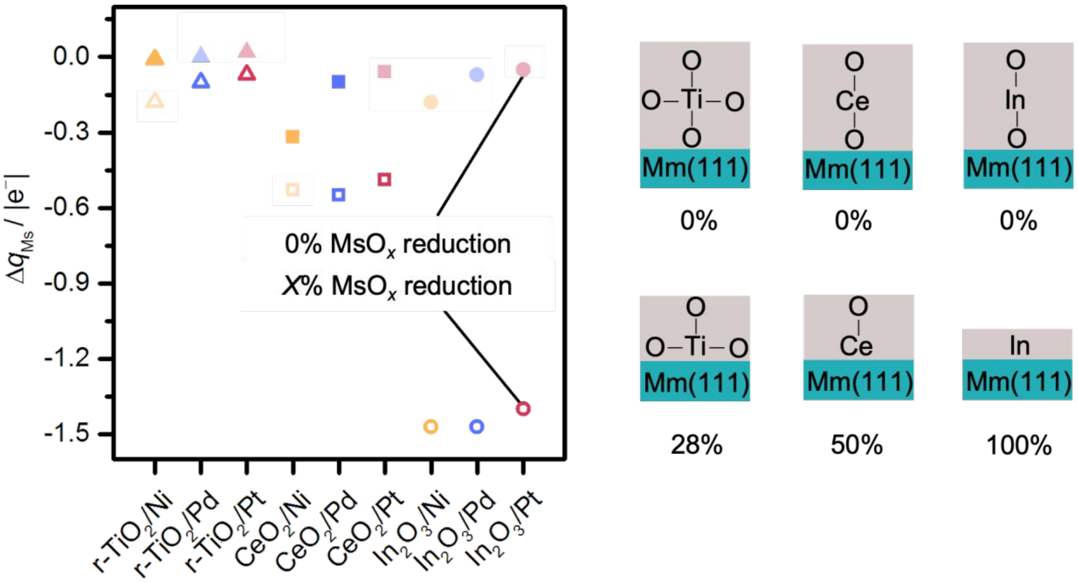

Figure S9. Electronic properties of Ti, Ce, and In atoms of the MsO_x at the interface with Mm(111) slabs. Δq_{Ms} is the shift in the average Bader charges of Ms in the MsO_x layers at different degrees of reduction interacting with Mm(111) slabs, with respect to that of the pristine isolated metal oxide layers. Filled and unfilled symbols indicate 0 and X% MsO_x reduction, respectively, where X% is indicated in the schemes. Semi-transparent data points represent structures that are not the most stable systems.

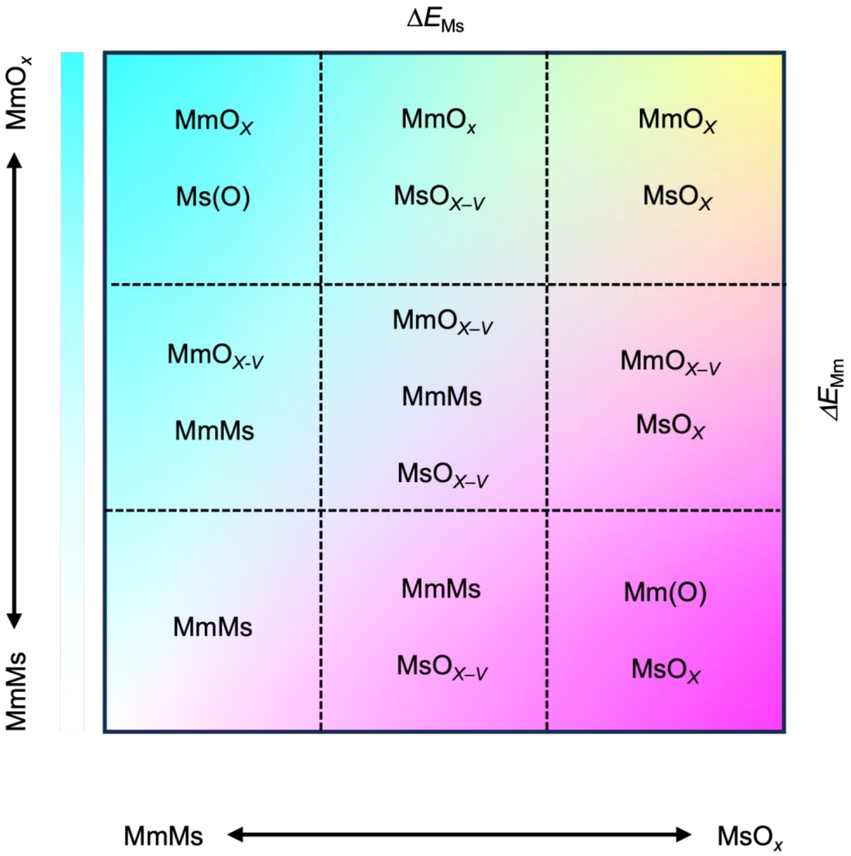

Figure S10. Phase diagram for classifying the architectures in different systems according to the formation of metal oxides or alloys. ΔE_{Ms} and ΔE_{Mm} are descriptors that represent the competition of each Ms and Mm to form a metal oxide or an alloy. Mm stands for Pd, Pt, and Ni, while Ms represents Ti, Ce, and In. Generally, in the region above the diagonal, Ms is more oxidized than Mm; on the contrary, Mm is more oxidized than Ms below the diagonal. Moreover, as the reaction conditions become more reductive, the alloy formation free energy is constant, but the free energy of oxide formation increases. Thus, considering the change in Gibbs free energy at different degrees of reductivity as a driving force for structural change, the boundaries of these areas will move toward right and upper (when the axes are based on potential energy). Therefore, the region of alloy formation gets larger, and the area of oxide formation becomes smaller.

4. Supporting References

(1) Albani, D.; Capdevila-Cortada, M.; Vilé, G.; Mitchell, S.; Martin, O.; López, N.; Pérez-Ramírez, J. Semihydrogenation of Acetylene on Indium Oxide: Proposed Single-Ensemble Catalysis. *Angew. Chem. Int. Ed.* **2017**, *56* (36), 10755–10760. https://doi.org/10.1002/ANIE.201704999.

(2) Capdevila-Cortada, M.; Vilé, G.; Teschner, D.; Pérez-Ramírez, J.; López, N. Reactivity Descriptors for Ceria in Catalysis. *Appl. Catal., B* **2016**, *197*, 299–312. https://doi.org/10.1016/J.APCATB.2016.02.035.

(3) Paunović, V.; Rellán-Piñeiro, M.; López, N.; Pérez-Ramírez, J. Activity Differences of Rutile and Anatase $TiO2_2$ Polymorphs in Catalytic HBr Oxidation. *Catal. Today* **2021**, *369*, 221–226. https://doi.org/10.1016/J.CATTOD.2020.03.036.

(4) Jain, A.; Ong, S. P.; Hautier, G.; Chen, W.; Richards, W. D.; Dacek, S.; Cholia, S.; Gunter, D.; Skinner, D.; Ceder, G.; Persson, K. A. Commentary: The Materials Project: A Materials Genome Approach to Accelerating Materials Innovation. *APL Mater.* **2013**, *1* (1), 011002. https://doi.org/10.1063/1.4812323/119685.

(5) Lide, D. R. *CRC Handbook of Chemistry and Physics*, 84th ed.; London, 1994.

(6) Stevanović, V.; Lany, S.; Zhang, X.; Zunger, A. Correcting Density Functional Theory for Accurate Predictions of Compound Enthalpies of Formation: Fitted Elemental-Phase Reference Energies. *Phys. Rev. B* **2012**, *85* (11), 115104. https://doi.org/https://doi.org/10.1103/PhysRevB.85.115104.

(7) Wang, X.; Beck, A.; van Bokhoven, J. A.; Palagin, D. Thermodynamic Insights into Strong Metal–Support Interaction of Transition Metal Nanoparticles on Titania: Simple Descriptors for Complex Chemistry. *J. Phys. Chem. A* **2021**, *9* (7), 4044–4054. https://doi.org/https://doi.org/10.1039/D0TA11650E.
